# Exploring Two Different Feeding Strategies During Dry Period With Regard to Dry Matter Intake, Intermediary Metabolism and Certain Health Aspects of Dairy Cows in Germany

**DOI:** 10.1111/jpn.14120

**Published:** 2025-04-22

**Authors:** Dana Carina Schubert, Kathrin Meetschen, Martin Pries, Sebastian Hoppe, Martin Holsteg, Hanna Rieger, Charlotte Vogel, Lothar Kreienbrock, Christian Visscher, Martin Höltershinken, Martina Hoedemaker, Josef Kamphues, Amr Abd El‐Wahab

**Affiliations:** ^1^ Institute for Animal Nutrition University of Veterinary Medicine Hannover, Foundation Hannover Germany; ^2^ Landwirtschaftskammer Nordrhein‐Westfalen, Versuchs‐ und Bildungszentrum Landwirtschaft Kleve Germany; ^3^ Institute for Biometry, Epidemiology and Information Processing University of Veterinary Medicine Hannover, Foundation Hanover Germany; ^4^ Clinic for Cattle University of Veterinary Medicine Hannover, Foundation Hannover Germany; ^5^ Department of Nutrition and Nutritional Deficiency Diseases Faculty of Veterinary Medicine Mansoura University Mansoura Egypt

**Keywords:** dairy cows, dry matter intake, dry period, feeding strategies, metabolism, milk production

## Abstract

Dry cow feeding plays an essential role in dairy production. Therefore, the objective of this study was to evaluate two different dry cow feeding strategies regarding dry matter intake (DMI), metabolism and health aspects of dairy cows in the first 50 days after calving. One hundred and six primiparous (*n* = 35) and multiparous (*n* = 71) cows (German Holstein) were assigned to one of two different feeding regimes 6–8 weeks before expected parturition. Group one phase (1P) received a single‐phase diet with 6.0 MJ NEL/kg DM. Group two phases (2P) received a two‐phase diet. During first 4–6 weeks of dry period, cows were fed a diet containing 5.5 MJ NEL/kg DM, while during the 2 weeks before expected parturition, cows received the transition diet (6.6 MJ NEL/kg DM). Post‐partum, all cows received the same ration (6.9 MJ NEL/kg DM) ad libitum. Considering the entire dry period, DMI was higher in the 1P multiparous (1P: 14.8 vs. 2P: 12.9 kg/day/animal, *p* < 0.001) and primiparous (1P: 12.0 vs. 2P: 9.90 kg/day/animal, *p* < 0.001) compared to 2P. During the whole trial, there were no differences in body weight and body condition score between treatments. No differences were observed in the serum levels of calcium, phosphorus, non‐esterified fatty acids (NEFA) and β‐hydroxybutyrate (BHB) between 1P and 2P multiparous. However, primiparous fed 1P showed higher serum NEFA levels during lactation period (1P: 611 vs. 2P: 425 µmol/L, *p* = 0.017) and higher BHB levels during preparation period compared to 2P (1P: 0.382 vs. 2P: 0.320 mmol/L, *p* = 0.016). The energy‐corrected milk yield (ECM) in multiparous showed no significant differences between feeding treatments (38.0 and 37.1 kg for 1P and 2P, respectively), while in the case of primiparous, the ECM yield differed between feeding systems (27.5 and 23.9 kg for 1P and 2P, respectively, *p* = 0.030). Results indicate that in our conditions, the additional effort associated with two‐phase feeding seems to have limited suitability for primiparous.

## 1 Introduction

High‐producing dairy cows live under quite heterogeneous nutritional and environmental conditions (Sundrum 2015). Cows' transition from the gestation period to lactation (3 week before to 3 week after calving), is the most challenging and critical period during the reproduction cycle in relation to the dairy cows' health status (Drackley 1999). Dry cow nutrition and management should aim to prepare the cows as best as possible for the next lactation. In this way, significant improvements in animal health and productivity can be achieved (Roche et al. 2013). After calving, the increased energy requirement in dairy cows for high milk production results in a negative energy balance in early lactation (Grummer et al. 2004; Goff 2006; Churakov et al. 2021). Throughout the previous few decades, nutritional strategies have changed. Traditionally, the dry period is separated into two feeding intervals. Cows are fed a high‐fibre, low‐energy diet during the ‘Far‐off’ period, which lasts for the first few weeks of the dry period (Gundelach et al. 2020). This is followed by a ‘Close‐up’ period during which cows are fed a diet with a higher energy density (Gundelach et al. 2020). The NRC (2001) recommends approximately 5.2 MJ NEL/kg of DM diet for far‐off dry period cows, and a 6.4–6.8 MJ NEL/kg of DM diet for ‘Close‐up’ cows, so as to allow the rumen and its microbes to adapt to the early lactation diet. This effect can also be achieved if the cows are fed a limited, and not ad libitum, higher energy diet (Drackley and Cardoso 2014). In general, over consumption of energy during the whole dry period compared with slightly restricted or need‐based energy supply seems to have detrimental effects on postpartal metabolism, mostly due to lower DMI and a more intense body fat mobilization (Drackley 2011; Drackley and Cardoso 2014). According to German feeding recommendations (DLG 2012), the two‐phase feeding of dry cows is considered most suitable for preparing the animals for early lactation. Nevertheless, in practice, the single‐phase system is common, especially with small herd sizes, which do not permit division into at least two further groups (due to lack of space). Furthermore, the two‐phase feeding is associated with a significantly higher workload, for example, due to the preparation of an additional ration and regrouping of the animals (Streuff et al. 2013).

A common recommendation to dairy farmers is to maximize DMI close to parturition to prepare the cows for a higher feed intake immediately after calving and, in turn, reduce metabolic disorders (Wathes et al. 2011; Esposito et al. 2014; Mellouk et al. 2019). A high DMI during the dry period can lead to significant increases in body fat which may depress appetite and thereby cause an increased incidence of periparturient disorders and impaired milk production (Rukkwamsuk et al. 1999). Energy intakes in excess of requirement, particularly during the early dry period, has been linked to reduced appetite/feed intake and an increased risk of health problems post‐partum (p.p.) according to several studies (Dann et al. 2006; Douglas et al. 2006; Janovick et al. 2011). There is circumstantial evidence that these negative effects are caused by the extensive mobilization of body mass (fat, protein) during the postparturient period (Dann et al. 2005; Janovick et al. 2011; McArt et al. 2012). Furthermore, when rations with higher energy density were fed during the prepartal period, DMI was found to be higher post partum (Grummer 1995; Rabelo et al. 2003). An increased energy density in the ‘Close‐up’ period may promote production and health in early lactation, as DMI is sharply reduced before calving (McNamara et al. 2003; Douglas et al. 2006; Graugnard et al. 2012; Vailati‐Riboni et al. 2019). Moreover, a 20%–40% gradual decline in DMI during the final 3 weeks of gestation (prefresh transition period) may initiate a negative energy balance and compromise the ability of dairy cows to adapt to physiological changes (Bell 1995; Grummer 1995; Grummer et al. 2004). In a study by Hayirli et al. (2002) who investigated data of 699 Holstein cows fed 49 different diets from 16 experiments conducted at eight universities, it was found that DMI decreased by about 32% during the final 3 weeks of gestation, and 89% of that decline occurred during the final week of gestation. Recently, Pérez‐Báez et al. (2019) observed that dairy cows start to decrease their DMI during the last 10 days of gestation, with a pronounced decrease during the last 3–4 days ante partum (a.p.). While it is true that substantial decreases of feed intake have been observed with diets including maize silage as one component during the close‐up period, the observed decreases have been considerably smaller with close‐up diets without maize silage (i.e., diets based on grass silage) (Agenäs et al. 2003).

Therefore, minimizing depression in DMI or increasing the nutrient density of the diet during the prefresh transition period is suggested to maintain body reserves, increase nutrients available for rapid fetal growth, udder development and production of colostrum, ease metabolic transition from pregnancy to lactation and acclimate rumen microorganisms to lactation diets (Nocek 1995; Hayirli and Grummer 2004; Girma et al. 2019). Factors affecting and regulating feed intake of lactating dairy cows are numerous and complex and span cellular to macro‐environmental levels (Roseler et al. 1997; Allen 2000). These factors may include animal factors (i.e., age, body condition, breed, physiological stage and milk yield level), dietary factors (i.e., ingredient and nutrient compositions of diets and physical and agronomic characteristics of feeds), management factors (i.e., production, feeding and housing systems) and climatic factors as temperature, humidity and wind (Hayirli et al. 2002; Grummer et al. 2004). Therefore, determination of factors affecting DMI and quantification of their effects are important for developing new feeding strategies being most suitable during the prefresh transition period. Zamet et al. (1979) found that DMI for cows that experienced health problems were 18% lower antepartum (a.p.) and 20% lower post partum (p.p.) than for healthy cows. In the study by Wallace et al. (1996), cows with any health disorder around parturition had decreased DMI during the first 20 days p.p. (13.9 vs. 17.8 kg/day). Hammon et al. (2006) and Huzzey et al. (2007) observed that cows developing uterine disease p.p. were affected by a reduced DMI beginning 1 week before parturition. These data indicated that predisposition to disorders at and immediately following parturition may be indicated by reduced DMI a.p. metabolic stress in early lactation related to negative energy balance and its correlation to health disorders is well documented (Pryce et al. 1998). However, there is a lack of information about metabolic stress during the dry period. The aim of the current study was to evaluate the advantages of two different dry cow feeding strategies, namely a single‐diet with medium energy content over the whole dry period (Group 1P) versus two‐phase diet with low‐energy ‘Far‐off’ ration and high energy ‘Close‐up’ ration (Group 2P) regarding DMI and intermediary metabolism as well as on possible periparturient health disorders or metabolic diseases of the dairy cows in the first 50 days after calving.

## 2 Materials and Methods

### 2.1 Animals and Housing

Primiparous and multiparous cows of the German Holstein breed from the Agricultural Research and Teaching Centre Haus Riswick of the Chamber of Agriculture in North Rhine‐Westphalia, Germany, were used in the present study from the beginning of the dry period or, in the case of primiparous, 8 weeks before the expected calving date until 50 days p.p. At the end of the study, the animals were returned to the normal dairy herd. The trial lasted a total of 9 months. The two experimental groups consisted of 63 animals each. The animals in each group were paired according to the number of lactations, expected dry period length (depending on calving interval), BW at the start of the trial (Group 1P: 717 ± 62.8 kg; Group 2P: 711 ± 73.0 kg), previous 305‐day milk yield or genetic merit for milk yield (primiparous) and first calving date (primiparous). The animals in each pair were then randomly assigned to the experimental groups. Each group was assigned approximately 28% primiparous (Group 1: *n* = 17; Group 2: *n* = 18), which equals the proportion of primiparous in the entire herd.

**Figure 1:**
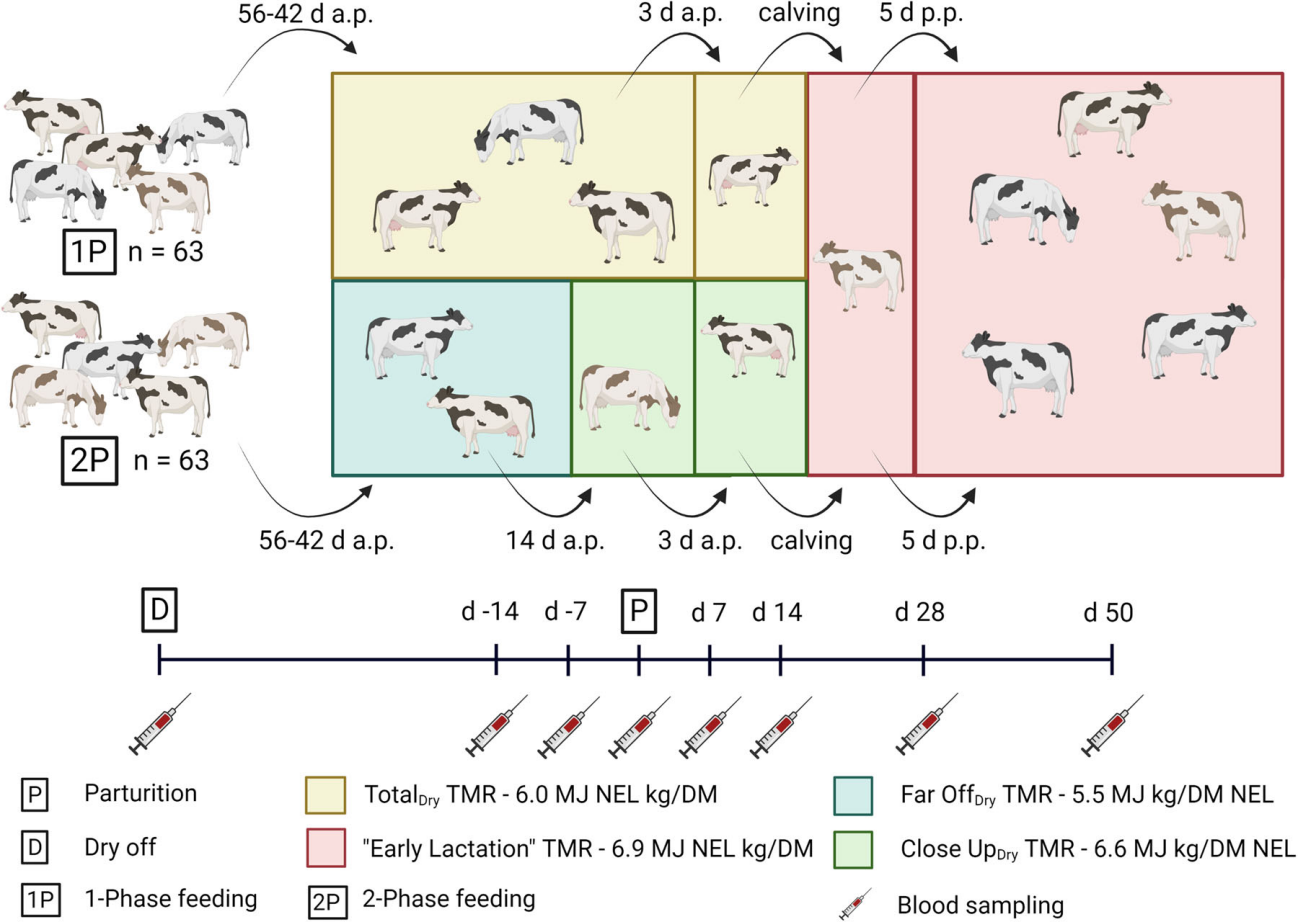
Scheme of the experimental set‐up and individual blood sampling times. Day—14: Day 14 antepartum (a.p.). Day 14: Day 14 post partum (p.p.). Total_Dry_: Total mixed ration (TMR) fed Group 1P during the entire dry period. Far Off_Dry_: TMR fed Group 2P during the dry period until Day 14 a.p. Drying off took place 49–42 days before the calculated calving date. Early Lactation, TMR fed both Groups from Days 1 to 50 p.p. Close Up_Dry_: TMR fed Group 2P during last 14 days a.p. 1P: single‐phase feeding; 2P: two‐phase feeding. Figure 1 was created with Biorender.com.

Dry cows were kept in a free stall barn with a slatted floor and two rows of raised cubicles with mattresses covered with straw. The two treatment groups were housed in separate compartments until calving (Figure 1). Animals in Group 2P were moved approximately 14 days (multiparous: 15.4 ± 6.26 days; primiparous: 11.3 ± 4.10 days) before the calculated calving date to an adjacent, separate area. Approximately 3 days before the expected calving date, the animals on both treatments were moved to straw yards. Immediately after calving, the cows were transferred to another deep litter pen, where the animals from both groups were mixed and were fed the same early lactation ration. After approximately 5 days, the cows from both treatments were moved to a free stall barn, receiving the early lactation diet until 50 days p.p.

Each stall barn provided the animals access to automatic water bowls and feeding troughs at all times. The weighing troughs (GEA Westfalia Separator GmbH, Oelde, Germany or custom‐made by Waagen Döhrn GmbH & Co. KG, Wesel, Germany) identified the cows either by means of transponders (GEA Westfalia Separator Group GmbH, Oelde, Germany) or chip cards (Waagen Döhren GmbH & Co. KG) and recorded feed intake parameters. Furthermore, pens were equipped with environmental enrichment material (cow brushes). Automatic animal scales were located at the exit of the milking parlour.

The lactating cows were milked twice a day in a 32‐unit rotary milking parlour (AutoRotor 32 Performer, GEA Farm Technologies GmbH, Bönen, Germany), with automatic milk quantity recording. The milking times started at 05:00 h and at 16:30 h. The freshly calved cows, which were still in the deep litter barn, were milked at the end of the milking period.

### 2.2 Rations and Feeding Regime

The cows were fed TMR ad libitum that were formulated to meet nutrient requirements for cows during the dry period and early lactation, respectively (NRC 2001). The chemical compositions for silages and ingredients are presented in Table 1. Overall, four different rations based on grass and corn silage were used during the trial (Table 2). Among them, three were fed during the dry period (Total_Dry_, Far Off_Dry_ and Close Up_Dry_) and one during early lactation (Early_Lac_). The nutrient and energy contents of the different rations are presented in Table 2. The Total_Dry_ TMR was fed to Group 1P and had an energy content of 6.0 MJ NEL/kg DM. Group 2P was fed two different diets according to the two‐phase feeding regime. During phase 1 (‘Far‐off’, first 4 to 6 weeks of dry period), cows received the Far Off_dry_ diet containing 5.5 MJ NEL/kg DM, while during phase 2 (‘Close‐up’), cows were fed the Close Up_Dry_ diet (6.6 MJ NEL/kg DM) for 2 weeks before the calculated calving date. However, during early lactation, both groups were fed the same TMR Early_Lac_ (6.9 MJ NEL/kg DM) directly after calving for the first 50 days p.p. The NRC (2001) recommendations are 5.2 MJ for the ‘far off’ and 6.4–6.8 MJ for the ‘close‐up’ ration as well as 7.5–6.7 MJ for fresh and early lactating cows, respectively. The experimental procedure scheme is displayed in Figure 1. Primiparous in the early preparation period assigned to the 2P system had a higher DCAD (2730 mEq/d) than those in the 1P system (2353 mEq/d; *p* = 0.001). During the late preparation period, 1P multiparous showed a higher nutrient intake (except crude fat and Ca) compared to those fed the 2P system. The DCAD was higher in 1P multiparous and primiparous during the late preparation period (2770 and 2308 mEq/d, respectively) compared to those in the 2P system (2134 and 1854 mEq/d, respectively).

**Table 1:** Chemical composition (g/kg DM) and energy (MJ/kg DM) of the roughages, wheat and rapeseed meal (extracted).

Parameter	Corn silage	Grass silage	Wheat	Post‐extraction rapeseed meal
DM (g/kg fresh)	381	428	868	882
Crude ash	36.0	101	20.0	81.6
Crude protein	64.8	155	139	377
Crude fat	29.5	38.6	21.0	44.1
Crude fibre	187	235	26.0	133
Starch	336	Not detected	706	12.0
Sugar	4.00	63.4	29.0	101
Ca	1.45	5.22		
P	1.90	3.80		
Na	0.022	1.61		
Mg	0.950	2.03		
K	9.32	27.2		
Cl	1.43	9.15		
S	0.895	3.47		
ME	11.2	10.6	13.4	11.9
NEL	6.83	6.36	8.60	7.24

**Table 2:** Ingredients, energy content and chemical composition of the TMR used for the experimental groups^a^ (1P and 2P) during dry period (TotalDry, Far OffDry and Close UpDry) and early lactation (EarlyLac).

	TMR and designated groups			
	1P	2P		1P and 2P
Item	Total_Dry_	Far Off_Dry_	Close Up_Dry_	Early_Lac_
Ingredient				
Grass silage, % DM	29.9	66.9	22.5	27.6
Corn silage, % DM	33.2	14.1	25.0	38.6
Wheat straw, % DM	19.5	18.1	14.5	2.00
Rapeseed meal, extracted, % DM	16.7	—	12.4	10.2
Wheat, % DM	—	—	12.2	—
Concentrates, % DM	—	—	12.6	20.7
Minerals and vitamins premix^b^, % DM	0.789	0.882	0.735	0.527
Limestone, % DM	—	—	—	0.395
Energy content and chemical composition				
DM, % FM^c^	45.0	52.0	41.7	46.1
ME, MJ/kg DM	10.0	9.35	10.8	11.3
NEL, MJ/kg DM	5.97	5.52	6.61	6.91
Crude protein, g/kg DM	134	111	146	155
Undegradable protein, g/kg DM	36.0	23.5	38.1	38.1
Utilizable crude protein, g/kg DM	139	119	151	158
Crude fat, g/kg DM	31.7	32.8	31.2	34.8
Crude fibre, g/kg DM	246	290	193	171
Crude ash, g/kg DM	72.9	92.6	63.2	61.7
Starch, g/kg DM	124	48.5	230	196
Total sugars, g/kg DM	23.5	14.8	33.3	52.1
NDF, g/kg DM	456	511	380	344
ADF, g/kg DM	282	308	228	207
NFC, g/kg DM	297	244	371	392
Calcium, g/kg DM	4.07	4.48	4.23	6.34
Phosphorus, g/kg DM	4.03	3.09	4.34	4.50
Sodium, g/kg DM	1.90	2.38	1.85	1.97
Magnesium, g/kg DM	3.36	3.17	3.39	2.54
Potassium, g/kg DM	16.4	23.0	13.5	14.8
Chloride, g/kg DM	5.54	9.33	4.79	4.93
Sulfur, g/kg DM	2.49	2.28	2.56	2.90
DCAD, mEq/kg DM	192	287	131	145

^a^ 1P: Single‐phase feeding; 2P: Two‐phase feeding.

^b^ Minerals and vitamins premix (Blattiviko Opti‐Chel, Höveler Spezialfutterwerke GmbH & Co. KG, Dormagen, Germany) contained per kg: 10 g zinc, 4.2 g manganese, 2.6 g copper, 0.15 g cobalt, 0.05 g selenium, 500,000 IU vitamin A, 50,000 vitamin D_3_, 10,000 vitamin E.

^c^ FM: Fresh matter, that is as fed. Total_Dry_ = 6–8 weeks a.p.; Far Off_Dry_ = 4–6 weeks; Close Up_Dry_ = 2 weeks a.p.

All diets were formulated and calculated in accordance with the guidelines of the NRC (2001). During the dry period, the energy requirement was calculated from the maintenance requirement and the requirement for pregnancy, udder development and maternal growth (e.g., heifers). During lactation, in addition to the maintenance requirement, the energy requirement necessary for milk production was also taken into account. The ration calculations were carried out with the help of the feeding software ‘Cattle 97’ (Rind 97, Chamber of Agriculture, North‐Rhine Westfalia, Germany). For the ration composition, the values of the individual components analyses before and during the trial were used as a basis for the main components (grass silage, corn silage, wheat). For the concentrate, supplementary feed and mineral feed, the declared values were used for the calculation. The rations were freshly mixed every morning by means of a self‐propelled mixer. In addition, cows had free access to drinking water.

### 2.3 Experimental Design, Sampling Procedures and Measurements

Multiparous entered the trial at the time of drying off, while primiparous were introduced into the trial on the basis of calculated calving date. In the case of primiparous, the term ‘preparation phase’ is used instead for dry period. Drying off of the multiparous took place continuously during the trial period, usually approx. 42–56 days before the calculated calving date or at a daily milk yield < 15 kg. For this purpose, the animals were taken from the group of late lactating cows, first milked completely in the milking carousel and then dried out. The drying off procedure included application of intrmammary antibiotic treatment (Benestermycin, Boehringer Ingelheim Vetmedica GmbH, Ingelheim, Germany; or Orbenin Extra, Zoetis Deutschland GmbH, Berlin, Germany) and use of an internal (Orbeseal, Zoetis Deutschland GmbH) and external mammary seal (ProfilacDryOff, GEA FarmTechnologies GmbH, Bönen, Germany). The primiparous were kept on pasture on the experimental farm before being included in the trial.

Before the start of the trial, silages were sampled using a silage drill. During the trial, samples were taken weekly from the cutting surface of the silages for chemical analysis. A representative sample was obtained from each new delivery of the concentrates and supplementary feeds. Three samples were combined and also chemically analyzed. A representative sample of the entire ration was taken daily in the morning to determine the DM content.

During the trial, feed intake (kg DM/d) was determined daily on individual animal basis by means of the weighing troughs that identified cows either by chip or transponder. Also, water intake was determined daily, but only during lactation. The daily duration of ruminating was recorded permanently over the whole trial in 48 randomly selected animals (1P: multiparous *n* = 19, primiparous *n* = 6; 2P: multiparous *n* = 17, primiparous *n* = 6) by means of a sensor (Heattime Pro System, SCR, Netanya, Israel) placed on the neck above the brachiocephalic muscle.

The BW was recorded weekly during dry period and daily during lactation period with the help of automatic animal scales (Taxatron 5000, GEA Farm Technologies GmbH). Furthermore, calves were weighed immediately after birth using cattle scales (Baumann Laufgewichtswaage, type 2002, Baumann Waagen‐ und Maschinenbau, Thiersheim, Germany; designed for BW up to approx. 300 kg, resolution 100 g). The body condition score (BCS), and backfat thickness (BFT) of both, dry and lactating cows, was assessed every 2 weeks. The classification of the BCS was graded according to the scheme developed by Edmonson et al. (1989). According to their BCS, the animals were classified on a scale from 1 (emaciated) to 5 (obese). The measurements of BFT were taken using an ultrasound device (Pavo Duo, Proxima Medizinische Systeme GmbH, Weil am Rhein, Germany) at the same time points as the BCS assessments. The measuring point was located between the middle and the rear third on an imaginary connecting line from the ischium to the hip bump (Stöber 2006).

From the beginning of drying off onwards, blood samples were taken at days 49, 14 and 7 before expected calving date, at the d of calving and then at days 7, 14, 28 and 50 p.p. Blood samples were collected from the *Vena jugularis externa* in serum‐tubes (10 mL, 95 × 16.8 mm, Sarstedt AG & Co., Nümbrecht, Germany), stored at room temperature until coagulation and then centrifuged for 10 min at 4000 rpm (Rotina 48, Firma Hettich GmbH & Co. KG, Tuttlingen, Germany). The serum was aliquoted in Eppendorf vials (2.0 mL, Sarstedt AG & Co. KG) and stored at −20°C until further analysis. Figure 1 shows a scheme of the experimental set‐up and the individual blood sampling times. In addition, all cows were clinically examined on the day of parturition and the 9 following days. Any diseases that occurred were documented. The frequency of milk fever was included in the evaluation of this study. This was diagnosed if the cows showed at least two of the typical symptoms of milk fever (i.e., inappetence, lowered temperature of extremities, grinding teeth, typical recumbent posture, muscle tremors, coordination disorders, inability to rise), whereas hypocalcaemia was defined as a calcium level in the serum below 2.1 mmol/L.

The individual milk yield was recorded at each milking time by the automatic milk metres of the milking carousel. Amounts of milk that were not suitable for human consumption (colostrum or during the waiting period after treatment) were noted by hand. At weekly intervals, individual milk samples were taken to determine the milk composition (fat [%], protein [%], lactose [%], urea [mg/L] and the cell content [1000/L]).

### 2.4 Analytical Methods

#### 2.4.1 Feed Analysis

All feed samples were analyzed by the laboratory of the Agriculture Communication and Service Company GmbH, Niederwiesa, Germany (LKS). The samples were prepared and analyzed by means of near‐infrared‐spectroscopy in accordance with the guidelines of the Association of German Agricultural Research and Research Institutes (VDLUFA) (Naumann and Bassler 2012).

#### 2.4.2 Measures in Milk

Analysis of milk samples was performed at the laboratory of the North Rhine‐Westphalia State Control Association (Dairy Performance Testing Department, National Control Association, Krefeld, Germany) in accordance with the Milk Quality Ordinance (§ 64 Food and Feed Code, LFGB). The fat, protein, urea and lactose contents were quantified using the fully automated Milkoscan FT analysis system (Foss GmbH, Hamburg, Germany) by means of infrared spectroscopy. The content of somatic cells in the milk was determined by a flow cytometric measurement (Fossomatic FC, Foss GmbH).

#### 2.4.3 Measures in Serum

Concentrations of calcium (Ca), phosphorus (P), NEFA and BHB were determined by photometric measurement. While Ca was analyzed by using the Cobas Mira automatic analyzer (Roche Deutschland Holding GmbH, Grenzach‐Wyhlen, Germany), P, NEFA and BHB were measured using a clinical chemistry analyzer (ABX Pentra 400, Horiba ABX SAS, Montpellier, France). Insulin and IGF‐1 were determined as described by Meyerholz (2014) by using an immunradiometric assay (IRMA; Insulin IRMA KIT, IM3210, Immunotech, Beckman Coulter Inc., CA, USA). The sensitivity of the method for insulin was 1.3 µIU/mL and intra‐ and inter‐assay coefficients of variance (%CV) were 6.0% and 8.0% respectively. For IGF‐1, the sensitivity of the assay was 2 ng/mL plasma/serum, and %CV for intra‐ and inter‐assays were 5% and 6% respectively.

### 2.5 Statistical Analysis

Statistical analyses were performed by using the statistical software SAS, version 9.4, TS level 1M5 (SAS Institute Inc., Cary, NC, USA). The data for the evaluation of the cell count appeared right‐skewed and therefore were log‐transformed to the base of 10 to hold the assumption for normality, which was checked by visual inspection of the model residuals. For the analysis of variance of the different target variables observed, a series of mixed models with repeated measurement in time were created for the different outcomes. The procedure GLIMMIX with animal as random variable and all other factors fixed with a compound symmetry covariance structure was applied.

As an example for this model approach, statistical evaluations of DM and nutrient intake, as well as water intake (excl. day of calving), BW (during lactation, daily measurements) and daily rumination time were performed using the following mixed model:

$$y_{\mathrm{ijkl}}=µ+{\mathrm{BEH}}_{i}+{\mathrm{VWO}}_{j}+{\mathrm{TAG}}_{k}+{\mathrm{BEH}}_{i}\times {\mathrm{TAG}}_{k}+{\mathrm{kuh}}_{l}+e_{\mathrm{ijkl}}.$$

The analysis of variance to test for influences on BCS, BFT, milk yield and milk composition were calculated as follows:

$$y_{\mathrm{ijkl}}=µ+{\mathrm{BEH}}_{i}+{\mathrm{VWO}}_{j}+{\mathrm{LWO}}_{k}+{\mathrm{BEH}}_{i}\times {\mathrm{LWO}}_{j}+{\mathrm{kuh}}_{l}+e_{\mathrm{ijkl}.}$$

The clinico‐chemical target variables (e.g., insulin and IGF‐1 concentrations) were analyzed using the following model:

$$y_{\mathrm{ijkl}}=µ+{\mathrm{BEH}}_{i}+{\mathrm{VWO}}_{j}+{\mathrm{kuh}}_{l}+e_{\mathrm{ijkl}},$$

where:

*y*_ijkl_ = observed target variable

*µ* = general expected intercept

BEH_*i*_ = fixed effect of feeding group

VWO_*j*_ = fixed effect of the experimental week

LWO_*k*_ = fixed effect of the lactation week

kuh_*l*_ = random effect of the individual animal

*e*_ijkl_ = random error

For the other target variables, models were developed of the same kind with no deeper check for additional interaction to hold the stability of model estimates. The models included the experimental week as effect of dry period length on one hand and the week of lactation on the other as this also effects among others milk yield, feed intake, body condition and blood variables independently.

Due to the explorative nature of the study, no multiple adjustment for statistical tests within the models were conducted and *p* values including some additional post hoc analyses were therefore considered as descriptive measures. The values in Tables 3, 4, 5, 6, 7, 8 were presented as least square mean (LSM).

**Table 3:** Relative DMI (% of BW) by multiparous and primiparous during the dry and lactation period depending on feeding (single‐ vs. two‐phase); results from multivariable linear model approach.

Type	WK a.p./p.p.	1P LSM	*n*	2P LSM	*n*	SED	*p* value
Multiparous dry period	−7	1.96	28	1.50	31	0.35	0.2251
	−6	2.11	44	1.66	39	0.12	0.0004*
	−5	2.02	32	1.61	43	0.11	0.0007*
	−4	1.92	38	1.71	41	0.11	0.0585
	−3	1.99	38	1.70	38	0.12	0.0151*
	−2	1.87	40	2.06	33	0.14	0.1889
	−1	1.66	24	1.87	22	0.22	0.3512
Multiparous lactation period	1	2.34	46	2.36	45	0.11	0.809
	2	2.81	46	2.98	45	0.13	0.189
	3	3.22	46	3.38	45	0.12	0.205
	4	3.35	46	3.55	45	0.13	0.115
	5	3.49	46	3.66	45	0.12	0.142
	6	3.67	46	3.80	45	0.12	0.243
	7	3.76	46	3.87	45	0.11	0.314
Primiparous whole preparation period	−7	1.77	10	1.20	10	0.19	0.0131*
	−6	1.79	15	1.37	12	0.14	0.0122*
	−5	1.84	16	1.45	14	0.19	0.0590
	−4	2.05	15	1.64	16	0.22	0.0765
	−3	2.14	14	1.60	18	0.23	0.0342*
	−2	1.91	13	1.76	16	0.35	0.6623
	−1	1.33	7	2.03	16	0.38	0.1040
Primiparous lactation period	1	2.45	10	2.46	15	0.11	0.900
	2	2.85	17	2.91	18	0.15	0.686
	3	3.08	17	3.10	18	0.18	0.871
	4	3.15	17	3.47	18	0.17	0.078
	5	3.27	17	3.58	18	0.15	0.048*
	6	3.32	17	3.71	18	0.25	0.121
	7	3.36	17	3.87	18	0.20	0.017*

Abbreviations: LSM = least square means, *n* = number of biological replicates, SED = standard error of the difference.

* Indicates a significant difference between the two treatments within each lactation type with regard to each dry period. 1P: Single‐phase feeding; 2P: Two‐phase feeding. *p* value for *t*‐test of the difference. Total dry period = 6–8 weeks a.p.; early dry period = 4–6 weeks; late dry period = 2 weeks a.p.

**Table 4:** Daily energy and nutrient intake of multiparous and primiparous during the early dry period (week 7–3 a.p.) and late dry period (2 week a.p.) depending on the feeding (single‐ vs. two‐phase); results from multivariable linear model approach.

Lactation type	Parameter		Early dry period				Late dry period		
		1P LSM	2P LSM	SED	*p* value	1P LSM	2P LSM	SED	*p* value
Multiparous	NEL (MJ/d)	90.8	69.5	2.42	< 0.001*	82.9	93.9	2.84	0.002*
	Crude protein (g/d)	2046	1408	54.1	< 0.001*	1872	2062	65.0	0.004*
	Crude fat (g/d)	481	405	12.7	< 0.001*	441	453	14.0	0.396
	Crude fibre (g/d)	3731	3495	101	0.021*	3459	2926	109	< 0.001*
	Starch (g/d)	1903	805	76.6	< 0.001*	1662	3056	113	< 0.001*
	Sugar (g/d)	359	202	11.3	< 0.001*	325	453	14.9	< 0.001*
	Ca (g/d)	61.6	55.0	1.78	0.004*	57.3	60.2	1.96	0.143
	P (g/d)	61.3	39.4	1.63	< 0.001*	56.4	61.0	2.01	0.024*
	K (g/d)	248	274	7.50	0.001*	234	210	8.10	0.004*
	DCAD (mEq/d)	2902	3381	88.4	< 0.001*	2770	2134	105	< 0.001*
Primiparous	NEL (MJ/d)	73.9	53.9	3.16	< 0.001*	68.2	67.9	3.12	0.922
	Crude protein (g/d)	1665	1093	70.5	< 0.001*	1537	1476	71.2	0.400
	Crude fat (g/d)	392	315	16.8	< 0.001*	365	338	14.7	0.087
	Crude fibre (g/d)	3040	2771	133	0.051	2879	2336	109	< 0.001*
	Starch (g/d)	1550	545	68.8	< 0.001*	1311	1963	150	0.002*
	Sugar (g/d)	292	151	12.8	< 0.001*	262	307	19.9	0.033*
	Ca (g/d)	50.3	42.9	2.23	0.002*	47.3	46.2	2.12	0.599
	P (g/d)	49.8	30.9	2.11	< 0.001*	46.2	43.5	2.21	0.229
	K (g/d)	202	218	9.15	0.084	194	172	8.50	0.012*
	DCAD (mEq/d)	2353	2730	110	0.001*	2308	1854	108	0.002*

Abbreviations: LSM = least square means, SED = standard error of the difference.

* Indicates a significant difference between the two treatments within each lactation type with regard to each dry period. 1P: single‐phase feeding; 2P: two‐phase feeding. *p* value for *t*‐test of the difference. Total dry period = 6–8 weeks a.p.; early dry period = 4–6 weeks; late dry period = 2 weeks a.p.

**Table 5:** Body weight (BW, kg), body condition score (BCS, score from 1 to 5) and back fat thickness (BFT, mm) of multiparous and primiparous during dry/lactation periods depending on feeding (single‐ vs. two‐phase); results from multivariable linear model approach.

Lactation type	Examination period	1P LSM	*n*	2P LSM	*n*	SED	*p* value
		BW					
Multiparous	Drying off	749	46	744	44	13.5	0.688
	Dry period	777	46	768	45	13.1	0.534
	Lactation period	692	46	680	45	11.7	0.301
Primiparous	Baseline	561	8	548	10	15.4	0.411
	Whole preparation period	615	17	612	18	31.0	0.814
	Lactation period	559	17	558	18	12.2	0.974
		BCS					
Multiparous	Drying off	3.58	32	3.67	29	0.12	0.452
	Dry period	3.83	46	3.71	45	0.09	0.228
	Lactation period	3.51	46	3.39	45	0.09	0.181
Primiparous	Baseline	3.58	8	3.77	13	0.20	0.373
	Whole preparation period	4.04	17	3.83	18	0.13	0.119
	Lactation period	3.63	17	3.59	18	0.14	0.743
		BFT					
Multiparous	Drying off	14.6	32	16.7	29	1.52	0.183
	Dry period	19.1	46	17.5	45	1.50	0.318
	Lactation period	16.3	46	14.5	45	1.28	0.177
Primiparous	Baseline	12.5	8	13.8	13	2.04	0.517
	Whole preparation period	15.3	17	13.7	18	1.39	0.280
	Lactation period	12.0	17	10.8	18	1.35	0.368

*Note:* 1P: Single‐phase feeding; 2P: Two‐phase feeding. *p* value for *t*‐test of the difference. Total dry period = 6–8 weeks a.p.; early dry period = 4–6 weeks; late dry period = 2 weeks a.p.; baseline: Sampling point when heifers were entering the study.

Abbreviations: LSM = least square means, *n* = number of biological replicates, SED = standard error of the difference.

**Table 6:** Calcium and phosphorus concentrations (mmol/L) in serum of multiparous and primiparous for different times/areas depending on the feeding in the dry period (single‐ vs. two‐phase); results from multivariable linear model approach.

Lactation type	Parameter	Examination period	1P LSM	*n*	2P LSM	*n*	SED	*p* value
Multiparous	Ca	Drying off day	2.50	46	2.51	45	0.03	0.832
		Dry period	2.44	46	2.46	45	0.03	0.491
		Calving day	1.97	46	1.84	44	0.08	0.104
		Lactation period	2.43	46	2.39	45	0.03	0.223
	P	Drying off day	1.41	46	1.61	45	0.06	0.001*
		Dry period	1.49	46	1.59	45	0.05	0.070
		Calving day	0.938	46	0.870	44	0.06	0.262
		Lactation period	1.23	46	1.16	45	0.04	0.086
Primiparous	Ca	Baseline	2.51	17	2.54	18	0.03	0.434
		Whole preparation period	2.49	17	2.39	18	0.03	0.011*
		Calving day	2.37	17	2.29	18	0.06	0.186
		Lactation period	2.49	17	2.51	18	0.04	0.662
	P	Baseline	1.81	17	1.74	18	0.08	0.337
		Whole preparation period	1.70	17	1.61	18	0.08	0.262
		Calving day	1.35	17	1.08	18	0.10	0.013*
		Lactation period	1.36	17	1.41	18	0.07	0.484

Abbreviations: LSM = least square means, *n* = number of biological replicates, SED = standard error of the difference.

* Indicates a significant difference between the two treatments within each lactation type with regard to sampling time. 1P: Single‐phase feeding; 2P: Two‐phase feeding. *p* value for *t*‐test of the difference. Total dry period = 6–8 weeks a.p.; early dry period = 4–6 weeks; late dry period = 2 weeks a.p. Baseline: Sampling point when heifers were entering the study.

**Table 7:** Non‐esterified fatty acids concentration (NEFA, µmol/L) and β‐hydroxybutyrate (BHB, mmol/L) in the serum of multiparous and primiparous depending on the feeding in the dry period (single‐ vs. two‐phase); results from multivariable linear model approach.

Lactation type	Parameter	Examination period	1P LSM	*n*	2P LSM	*n*	SED	*p* value
Multiparous	NEFA	Drying off day	214	46	216	45	23.5	0.950
		Dry period	260	46	225	45	28.7	0.225
		Calving day	994	46	834	44	85.0	0.065
		Lactation period	700	46	738	45	65.3	0.568
	BHB	Drying off day	0.401	46	0.400	45	0.03	0.975
		Dry period	0.354	46	0.386	45	0.02	0.098
		Calving day	0.479	46	0.459	44	0.04	0.627
		Lactation period	0.732	46	0.742	45	0.07	0.891
Primiparous	NEFA	Baseline	463	17	397	18	79.0	0.408
		Whole preparation period	323	17	314	18	39.4	0.822
		Calving day	979	17	789	18	117	0.122
		Lactation period	611	17	425	18	73.6	0.017*
	BHB	Baseline	0.456	17	0.440	18	0.05	0.727
		Whole preparation period	0.382	17	0.320	18	0.03	0.016*
		Calving day	0.493	17	0.432	18	0.06	0.313
		Lactation period	0.535	17	0.466	18	0.04	0.102

Abbreviations: LSM = least square means, *n* = number of biological replicates, SED = standard error of the difference.

* Indicates a significant difference between the two treatments within each lactation type with regard to sampling time. 1P: Single‐phase feeding; 2P: Two‐phase feeding. *p* value for *t*‐test of the difference. Total_Dry_ = 6–8 weeks a.p.; far Off_Dry_ = 4–6 weeks; close Up_Dry_ = 2 weeks a.p. Baseline: Sampling point when heifers were entering the study.

**Table 8:** Milk yield and milk composition of multiparous (1P: *n* = 290; 2P: *n* = 279) and primiparous (1P: *n* = 106; 2P: *n* = 117) in the lactation period (data collected up to the 50th day of lactation); results from multivariable linear model approach.

Lactation type	Parameter	1P LSM	2P LSM	SED	*p* value
Cow	Milk yield (kg/d)	37.5	37.0	1.22	0.704
	ECM (kg/d)	38.0	37.1	1.24	0.488
	Fat content (%)	4.21	4.11	0.10	0.308
	Protein content (%)	3.29	3.23	0.05	0.201
	Lactose content (%)	4.72	4.72	0.02	0.833
	Urea (mg/L)	133	131	2931	0.999
	Cell count (10^3^/mL)	48.1	63.8	0.23	0.217
Heifer	Milk yield (kg/d)	27.3	24.5	1.41	0.050
	ECM (kg/d)	27.5	23.9	1.58	0.030*
	Fat content (%)	4.09	4.00	0.18	0.621
	Protein content (%)	3.30	3.33	0.07	0.658
	Lactose content (%)	4.87	4.84	0.05	0.535
	Urea (mg/L)	155	149	10.4	0.592
	Cell count (10^3^/mL)	39.6	63.7	0.30	0.122

Abbreviations: LSM = least square means, *n* = number of observations, SED = standard error of the difference.

* Indicates a significant difference between the two treatments within each lactation type. 1P: Single‐phase feeding; 2P: Two‐phase feeding. *p* value for t‐test of the difference. Total dry period = 6–8 weeks a.p.; early dry period = 4–6 weeks; late dry period = 2 weeks a.p.

## 3 Results

### 3.1 Feed and Water Intake

#### 3.1.1 DMI

**Figure 2:**
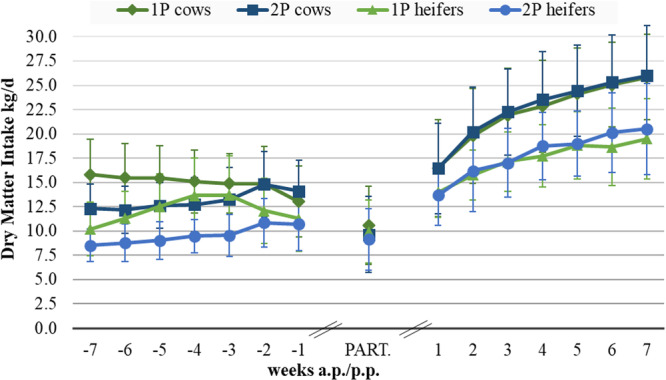
Dry matter intake (kg DM/day) during peripaturient period of multiparous and primiparous depending on feeding in the dry period (single‐ [1P] vs. two‐phase [2P]).

DM intake at early dry period and preparation period, respectively, was higher in 1P than in 2P, for both multiparous and primiparous (*p *< 0.001, Figure 2, Table S2), while the DMI in the late dry period did not display marked differences depending on feeding regime, neither in multiparous (*p* = 0.270) nor in primiparous (*p *= 0.102). However, the duration during which the close‐up ration was fed differed from the planned duration and lasted 15.4 ± 6.26 days for the multiparous and 11.3 ± 4.10 days for the primiparous (see Table S1). Considering the entire dry period, the DMI was still higher in the single‐phase fed multiparous (*p* < 0.001) and primiparous (*p *< 0.001) compared to two phase feeding (Figure 2, Table S2). At day of parturition, the DMI did not differ depending on feeding, neither in multiparous nor in primiparous. This was also the same for the lactation period in multiparous (*p* = 0.509) and in primiparous (*p *= 0.151). The relative DMI (kg DM/100 kg BW) in the dry period and lactation was calculated (Table 3). There were differences in the relative DMI during the dry period between 1P and 2P feedings in certain wk before calving in the multiparous (week—6: *p* = 0.004; week—5: *p* = 0.007 and week—3: *p* = 0.015) and in primiparous (week—7: *p* = 0.013; week—6: *p* = 0.012 and week—3: *p* = 0.034). During the whole lactation period, the relative DMI of the multiparous did not differ significantly between treatment groups (1P and 2P). This trend was also evident in the primiparous, except at Week 5 (*p* = 0.048) and Week 7 (*p *= 0.017) where the relative DMI of group 2P was higher than that of those animals with 1P feeding (Table 3).

#### 3.1.2 Water Intake

For multiparous, there were no significant differences in water intake during lactation between treatment groups 1P and 2P (1P: 92.9 kg/day/animal; 2P: 92.9 kg/day/animal; *p* = 0.990). Similarly, no significant differences in the water intake during lactation for primiparous between treatment groups were found. Water intake of the primiparous was 73.0 and 72.3 kg/day/animal for 1P and 2P, respectively (*p* = 0.860).

#### 3.1.3 Nutrient Intake

During the early preparation period, multiparous following the 1P system showed a higher nutrient intake compared to those fed the 2P system (Table 4). However, the DCAD was higher in cows following the 2P system (3381 mEq/d) compared to those in the 1P system (2902 mEq/d; *p* < 0.001). Furthermore, during the early preparation period, primiparous in the 1P system showed a higher intake of nutrients (except for crude fat and potassium) compared to those in the 2P system. Furthermore, during the late preparation period, primiparous in the 1P system showed a higher nutrient intake (except in NEL, CP, crude fat, Ca, P) compared to those in the 2P system.

### 3.2 Rumination Duration

**Figure 3:**
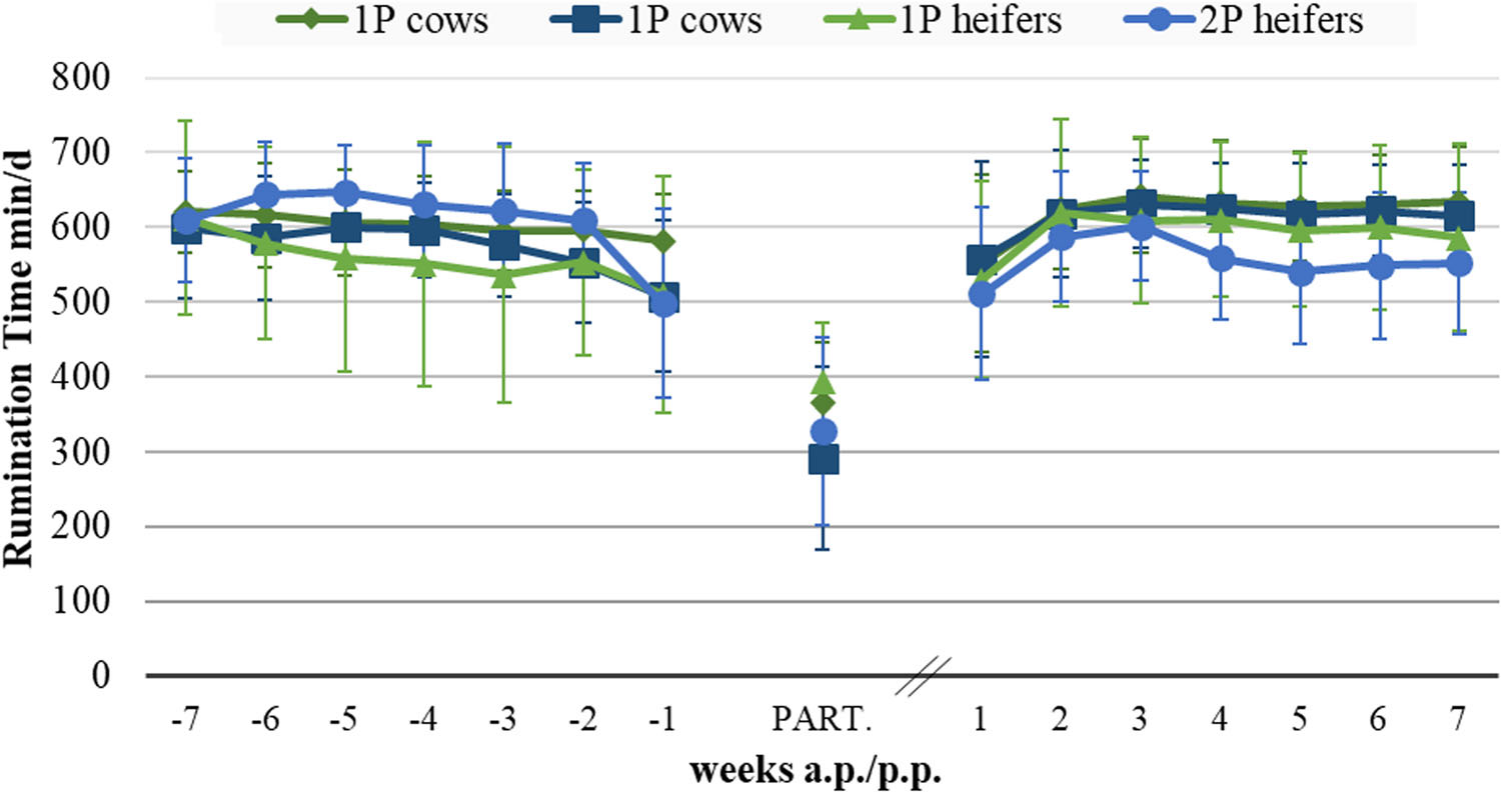
Daily rumination time (min) of multiparous and primiparous during peripaturient period (7 weeks ante partum to 7 weeks post partum) depending on feeding in the dry period (single‐ [1P) vs. two‐phase [2P]).

The mean daily rumination times of the multiparous and heifers of both feeding regimes varied over the weeks of the trial, ranging between around 500–600 min/animal (Figure 3, Table S3). During the early dry period, no significant differences in rumination time were noted in multiparous fed either 1P or 2P. However, differences in rumination time were observed in multiparous between the 1P and 2P feeding systems during the late dry period (591 and 524 min/animal, respectively, *p* = 0.001) and total dry period (603 and 574 min/animal, respectively, *p* = 0.046). At the day of calving as well as during the lactation period, multiparous showed no significant differences in the daily rumination time between both feeding systems, while in the case of primiparous, no significant differences were found in the rumination time between feeding systems at any examination period.

### 3.3 Body Weight, Body Weight Changes, Body Condition Score and Back Fat Thickness

At the time of drying off, the BW of multiparous in both treatment groups were on the same level (1P: 749 kg vs. 2P: 744 kg; Table 5). Over the entire dry period of cows, there were no significant differences in BW between the treatment groups (*p* = 0.534). Moreover, during the lactation period of multiparous, the differences in BW were not statistically significant between treatment groups (*p *= 0.301). Similarly, the BW of the primiparous showed no significant differences between the two treatment groups during the preparation or lactation period (Table 5). Body weight loss was calculated as the difference from average weight of each animal in first week of lactation and seventh week of lactation. Multiparous did not differ regarding weight loss during lactation (1P: −33.7 ± 31.4 kg BW; 2P: −34.1 ± 35.0 kg BW), whereas primiparous of group 2P lost less BW than 1P (1P: −22.8 ± 21.9 kg BW; 2P: −7.28 ± 21.6 kg BW). There were no significant differences regarding BCS and BFT either during the entire study between treatment groups for multiparous and primiparous.

### 3.4 Clinico‐Chemical Measures in Serum

#### 3.4.1 Calcium and Phosphorus Concentrations

The Ca concentration in the serum of all animals at the day of drying off (multiparous) or entering the study (primiparous), regardless of the lactation type and treatment group, was 2.5 mmol/L (Table 6). With regard to the serum Ca concentration of multiparous, there were no significant differences at any of the examination periods. However, Ca levels in the serum of primiparous differed between feeding systems during the preparation period (2.49 vs. 2.39 mmol/L for 1P and 2P, respectively; *p* = 0.011). The Ca concentration in the serum of the primiparous did not differ significantly on the day of calving or during lactation period between 1P and 2P feeding. At the time of drying off, the multiparous following the 2P system showed a higher P content in the serum compared to those of the 1P system (1.61 and 1.42 mmol/L, respectively; *p* = 0.001). However, the P concentration in the serum of multiparous did not differ significantly between feeding systems during further examination periods. In primiparous, the serum P concentrations differed only at calving d between the two feeding systems (1.35 vs. 1.08 mmol/L; *p* = 0.013). Nonetheless, no other differences were found regarding serum P content between both groups during other examination periods.

#### 3.4.2 Non‐Esterified Fatty Acids and β‐Hydroxybutyrate Concentrations

The NEFA and BHB concentrations in the serum of multiparous did not differ significantly between feeding systems (1P and 2P) within each examination period (Table 7). During other examination periods (except for lactation period), the NEFA contents in serum of primiparous did not differ significantly. Moreover, the BHB concentrations in the serum of primiparous differed only during the whole preparation period between feeding systems (0.382 and 0.320 µmol/L for 1P and 2P, respectively; *p* = 0.016), while during other examination periods, the BHB contents in the serum of primiparous did not differ statistically significantly.

#### 3.4.3 Hypocalcaemia and Hyperketonaemia

In general, it was observed that the number of multiparous and primiparous with clinically apparent milk fever did not differ significantly in the 1P and 2P systems (25.4% vs. 37.1%, respectively, *p* = 0.158, data not shown). Moreover, according to laboratory results, it was observed that the proportion of animals that were affected by hypocalcaemia (≤ 2.1 mmol/L) at the day of calving did not differ between the two systems (42.9% and 58.1% for 1P and 2P, respectively). The share of cows and primiparous with BHB concentrations in serum ≥ 1.2 mmol/L during the early lactation period (until 50 days p.p.) did not differ significantly between the two systems, 1P and 2P (17.5% vs. 14.5%, respectively; *p* = 0.6535). In terms of type and frequency of other postpartal diseases and the incidence of dystocia, the groups did not differ either.

#### 3.4.4 Insulin and Insulin‐Like‐Growth Factor (IGF‐1)

**Figure 4:**
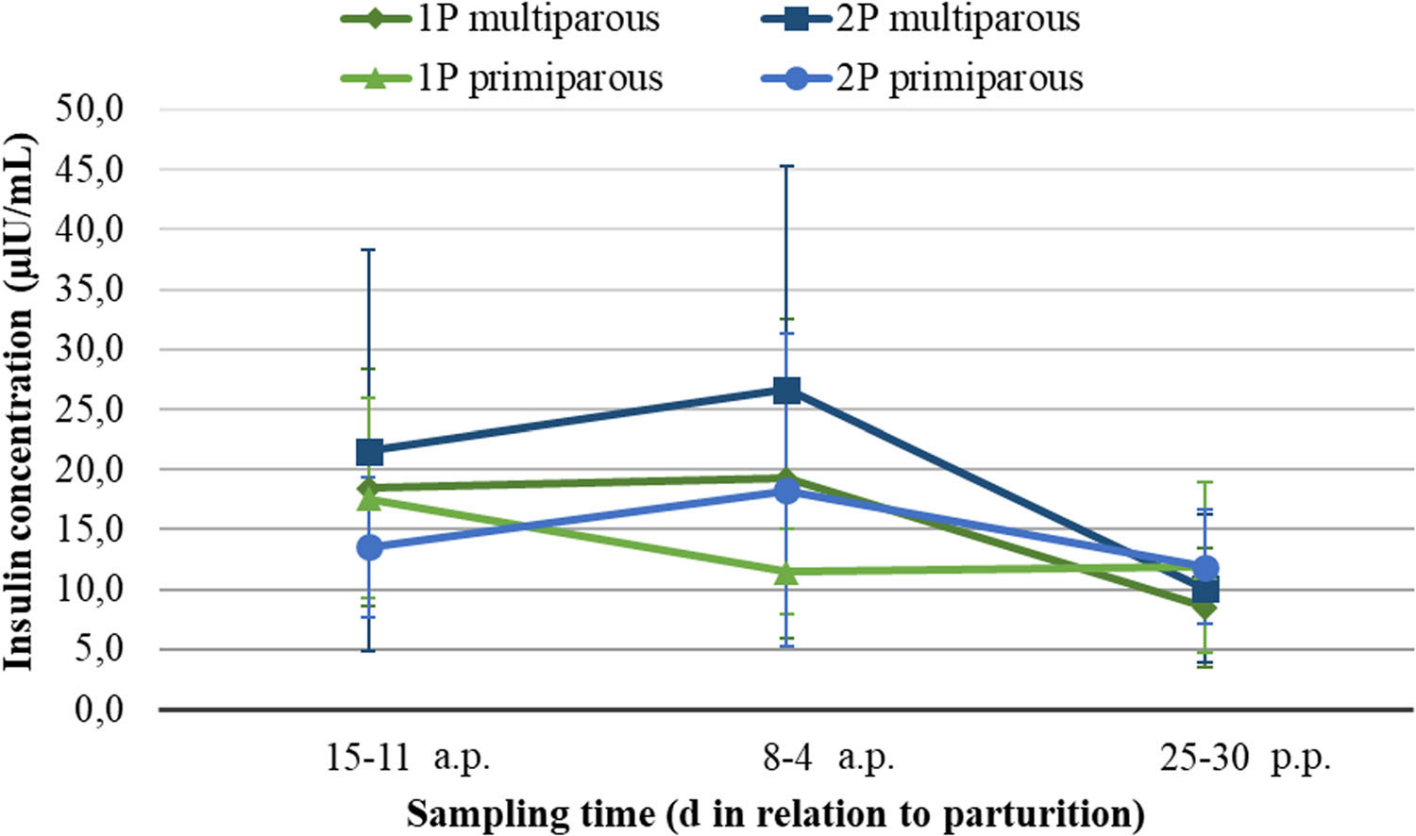
Insulin concentration (ng/mL) in the serum of multiparous and primiparous in different sampling periods depending on feeding during the dry period (single‐ vs. two‐phase); results from multivariable linear model approach.

**Figure 5:**
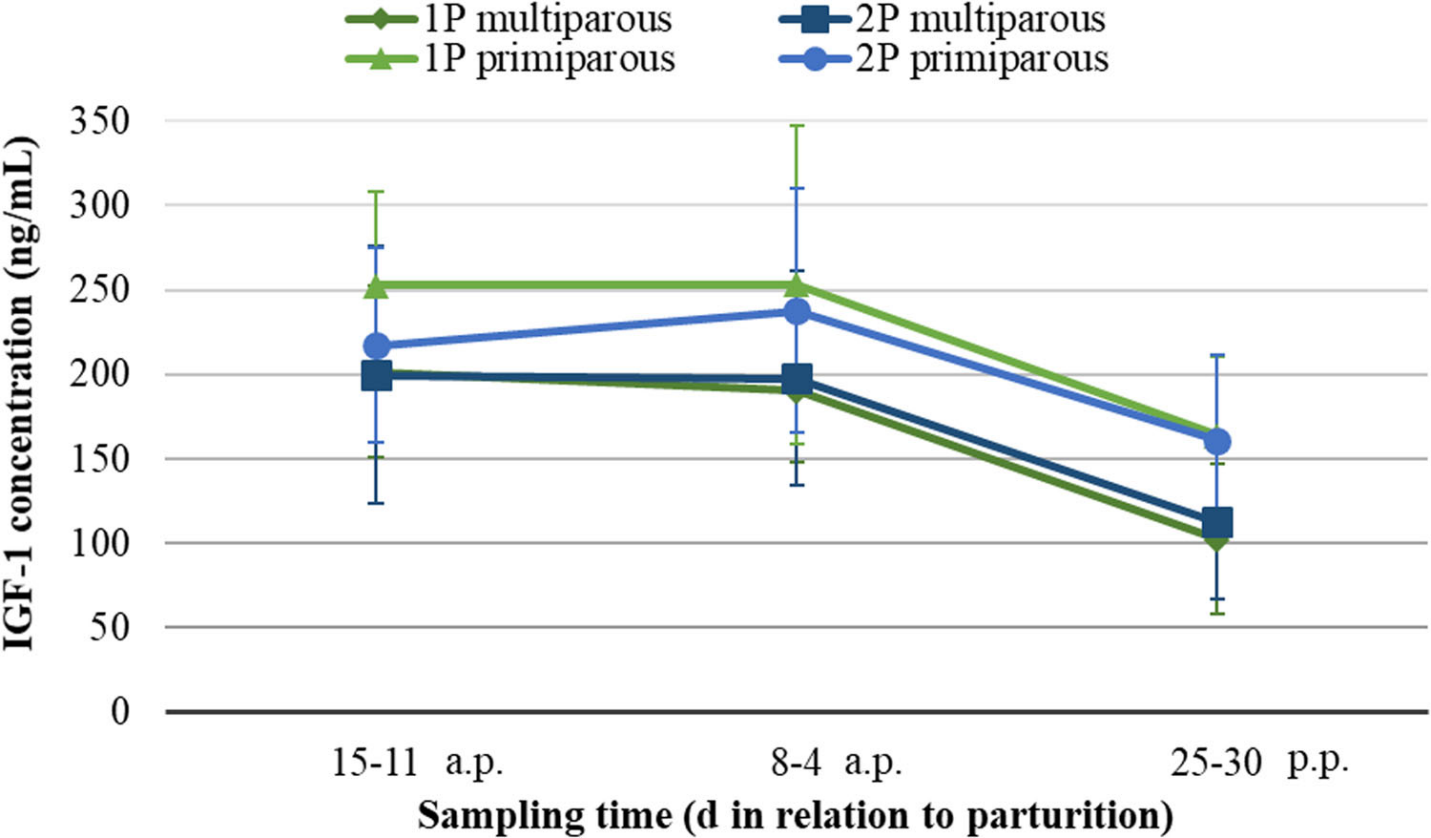
IGF‐1 concentration (ng/mL) in the serum of multiparous and primiparous in different sampling periods depending on feeding during the dry period (single‐ vs. two‐phase); results from multivariable linear model approach.

In multiparous and primiparous, the effect of the feeding system, either 1P or 2P, did not affect the insulin content in serum regardless of the time of sampling (a.p. or p.p.; Figure 4). However, insulin concentrations seemed to be decreased after calving, however it was not statistically tested (from 19.7 (a.p.) to 7.26 (p.p.) and from 22.4 (a.p.) to 9.87 (p.p.) µIU/mL for 1P and 2P, respectively). Similarly, no significant differences were observed in the IGF‐1 concentration in serum of multiparous and primiparous fed either 1P or 2P (Figure 5).

### 3.5 Milk Yield and Milk Composition

The milk yield did not differ significantly between the two treatment groups for multiparous (*p* = 0.704). The difference in the milk yield of the primiparous between the two treatment groups was around 2.8 kg/day/animal (*p *= 0.051; Table 8). The ECM yield in multiparous showed no significant differences between feeding systems (38.0 and 37.1 kg, for 1P and 2P, respectively), while in the case of primiparous, the ECM yield differed between feeding systems (27.5 and 23.9 kg/day/animal, for 1P and 2P, respectively; *p *= 0.030). The different feeding strategies in the dry/preparation period did not result in any changes in milk composition in either multiparous or primiparous (Table 8).

## 4 Discussion

The aim of the present investigation was to compare a single and a biphasic feeding concept for dry cows with regard to feed, energy and nutrient intake as well as metabolic health of the animals in the dry period and early lactation (up to the 50th day of lactation).

The shifting to the ‘Close‐up’ ration appeared to be problematic in the current study, as especially primiparous calved considerably earlier than the calculated calving day, which resulted in a shorter close‐up period than planned. In general, the available literature suggest that the higher‐energy ration should be fed 2–3 weeks before calving (Mashek and Beede 2001; Corbett 2002; Contreras et al. 2004). Gundelach et al. (2020) also chose to provide the close‐up ration for the last 2 weeks a.p. but do not report about deviations regarding planned and actual duration of this feeding phase. Richards et al. (2020) chose to switch to the close‐up ration 21 days before calculated calving, which resulted in an actual period of 19 ± 6 days on the close‐up ration. This suggests that deviations from the calculated date of birth might have been less significant overall if the ration had been given over a longer period. It might therefore be advisable to provide the ‘Close‐up’ ration for the last 3 weeks before the calculated calving date instead of 2 weeks. Additionally, it should be stated, that the 2P feeding was accompanied by additional moving and thus potentially additional stress, whereas this was not the case with the 1P cows. However, also under non‐experimental conditions, 2P feeding is associated with additional rehousing, so that the experimental conditions largely corresponded to reality in this respect. Furthermore, it should be mentioned that the starch level in the close‐up ration was increased to promote the development of the ruminal papillae, which are required for adequate absorption of the volatile fatty acids produced during rumen fermentation (Overton and Waldron 2004).

### 4.1 Feed Intake and Body Condition

While the single‐phase feeding strategy resulted in higher DMI during the early dry/preparation period, the feed intake did not differ in the late dry/preparation period, but was still higher in 1P cows and heifers considering the entire dry/preparation period. This finding goes in line with previous studies, which reported higher prepartal DMI in cows fed a higher energy ration during the dry period (Holcomb et al. 2001; Rabelo et al. 2003; Drackley and Cardoso 2014). Contradictorily, Gundelach et al. (2020) could not prove any differences in DMI of dairy cows depending on single‐ or two‐phase feeding. Regarding effects of energy density in the ration fed prepartum on DMI in early lactation, the literature provides contradictory results. While some studies, including the present one, found no effect of energy level in the prepartal diet on DMI p.p. (Holcomb et al. 2001; Gundelach et al. 2020), there are findings that suggest an increase in DMI due to higher energy levels in the diet during early lactation (Rabelo et al. 2003). Variable findings could be due to the fact that the studies differed both in the absolute energy densities of the diets used and in the difference between the energy densities of the rations used.

Independently of the feeding regime, there were marked differences in the DMI between primiparous and multiparous both, during preparation/dry period and lactation period, as observed by many earlier studies (Jensen et al. 2015). The lower DMI of primiparous is caused by the different body weight (size, frame) and—during lactation—by the lower milk production of primiparous. In equations to predict the DMI of dairy cows, the number of lactation, the body weight, the daily milk yield and the time (days in lactation) are the most important factors that determine the DMI (Gruber et al. 2004; NASEM National Academies of Sciences Engineering and Medicine 2021; GfE Society of Nutrition Physiology 2023). It has to be underlined that also the digestibility of the ration is very important. Thus, it is not astonishing that the primiparous animals of 2P had the lowest DMI in the first weeks of the preparation/dry period when the ration with the lower energy density (due to lower digestibility) was fed.

It was indicated that DMI and energy intake during the dry/preparation period were higher in 1P than in 2P multiparous and primiparous. In terms of BFT, the multiparous and primiparous of both groups in this study remained considerably below the reference values of 22–23 mm for cows at drying off and of 20–30 mm for cows at parturition by Schröder and Staufenbiel (2003). On the contrary, multiparous and primiparous could be considered obese according to BCS. Only 2P primiparous (3.72 BCS points) were in the recommended range of 3.25–3.75 at the last measurement before calving according to Mansfeld et al. (2000), while the other groups exceeded this recommendation (1P multiparous: 3.96; 1P primiparous: 4.16; 2P multiparous: 3.84). A BCS above 3.5 points was considered a risk factor for more severe p.p. NEB and resulted in body reserve mobilization and associated impaired liver function (Garro et al. 2014). In the present study, no differences between treatment groups in BW, BCS or BFT were observed. However, reasons why the increase in BFT and BCS during the dry period was numerically greater in group 1P than in group 2P, while BW barely differed, remain unclear. In general, there is good correlation between BW, BCS and BFT. Nevertheless, Berry et al. (2006) found that differences in BCS approximately account only for 30% of the variation in BW. According to Schröder and Staufenbiel (2003) 1 mm difference in BFT equals to 5 kg of total body fat, but differences in BW do not only result from changes in body fat, but also due to muscle loss/growth, reproduction cycle, level of gut fill or age‐dependent growth (Enevoldsen and Kristensen 1997; Koenen et al. 1999).

### 4.2 Clinico‐Chemical Measures in Serum

The beginning of lactation poses an enormous challenge for dairy cows in terms of maintaining calcium homeostasis and is associated with a high risk of developing milk fever (DeGaris and Lean 2008). Regulatory mechanisms for rapid mobilization of calcium for milk production can be promoted during the dry period by feeding measures, for example, limited calcium intake or availability (Goff 2006; Aubineau et al. 2022). In the current study, Ca content of the feed was comparable for both groups. Yet, as DMI was higher during dry period in 1P cows, accordingly also Ca intake was higher in 1P than in 2P multiparous. This did not result in significant differences in the cows' calcium levels or a higher incidence of milk fever. Primiparous are less prone to developing hypocalcaemia or milk fever (DeGaris and Lean 2008; Venjakob et al. 2017; Lean et al. 2023). Therefore, the significantly lower serum Ca levels in the biphasic fed primiparous compared to 1P heifers during the preparation period can be considers as not relevant from a biological point of view. Additionally, the measured values were within the reference range of 2.0–3.0 mmol/L given by Stöber and Gründer (2012). However, the lower DMI and thus the correspondingly lower uptake of Ca in 2P primiparous during the early preparation period could be the cause of an overall lower Ca level in the blood during the entire dry period. There may be a disadvantage here for the heifers, which are still growing, or also for fetal growth, since Ca as a mineral is of great importance, especially with regard to the mineralization of the skeleton (Erben 2015).

During the dry period, NEFA concentrations did not differ between 1P and 2P multiparous and between 1P and 2P primiparous. In addition, the NEFA concentrations of all groups were below the upper reference value of 360 µmol/L given by Stöber and Gründer (2012) who only provide one reference value for the entire peripartal period. According to Fürll (2013), however, the values measured a.p. should be below 150 µmol/L and were therefore too high in all groups. In agreement with the results of Graugnard et al. (2012), who described an increase in NEFA concentrations on the day of calving regardless of the dietary supply (high/low energy) a.p., NEFA levels in the present study clearly increased in all groups at the day of calving. However, this increase was more pronounced in the 1P cows than in the 2P cows, which resulted in a tendency towards higher values at the d of calving. Doepel et al. (2002) also found that a higher energy density in the ration during 3 weeks before calving was associated with lower NEFA concentrations at the d of calving. In contrast, Dann et al. (2006) found that cows with a lower energy supply during the ‘Far off’ period had lower serum NEFA concentrations compared to cows that were supplied with 150% of NRC recommendations during far‐off, while there were no effects of different energy levels during the close‐up period. The authors concluded that metabolism peripartum was especially affected by overfeeding during far‐off rather than by differences in ‘close‐up’ treatments (Dann et al. 2006). In agreement with Dann et al. (2006), who reported no residual effects in early lactation, there was no significant difference in NEFA levels between the multiparous during lactation in the present study. By contrast, the 1P primiparous had higher NEFA concentrations than the 2P primiparous during lactation. This finding could be explained by a marked drop in BCS from 4.04 (preparation period) to 3.63 (lactation period) in 1P primiparous, as mobilization of body fat increases plasma NEFA levels (Lacetera et al. 2005; Barletta et al. 2017). On the other hand, ECM yield was higher in the 1P primiparous than in the 2P primiparous, and a higher milk yield leads to higher NEFA concentrations depending on the energy balance (Hart et al. 1978). In terms of BHB concentrations in the serum, 1P and 2P multiparous did not differ throughout the trial, and also relating the incidence of hyperketonaemia (≥ 1.2 mmol/L), there was no significant difference between the treatment groups in either the multiparous or the primiparous. Highest BHB concentrations were obtained during early lactation in both treatment groups. The reduced DMI in the peripartal period as well as the high milk yield at the beginning of lactation in all groups and the resulting negative energy balance in the present trial thus explain the increased BHB serum levels at the beginning of lactation (Baumgartner 2009).

Serum measures were evaluated to obtain more detailed information regarding metabolic stress and health status. The daily rumination time as an ‘indicator’ for the well‐being of the animals (Rosenberger et al. 2012) was also comparable in this study within the respective lactation classes in early lactation, whereby calving itself had a considerable effect on the rumination time in all groups, but not the previously practiced ration design. Therefore, concerns that the single‐phase feeding is associated with an increased risk of health disorders in the peripartal period (Drackley et al. 2005; Drackley and Guretzky 2007; Drackley 2011; Streuff et al. 2013) was not confirmed by the present study. Nevertheless, the overall incidence of disease in the present study was quite high, with about two‐thirds of all animals diagnosed with at least one disorder in the peripartal period. According to LeBlanc (2010), up to one‐third of dairy cows is affected by metabolic or infectious disorders during early lactation, while Piechotta et al. (2012) also found at least one p.p. disease in approx. 66% of the animals with comparable intensive veterinary care/animal health control. In the present trial, animal control was very intensive, especially during the first 10 days of lactation when a veterinary general examination was carried out daily. Therefore, it was likely to result in a very high proportion of affected animals being recognized as such.

### 4.3 Milk Yield and Milk Composition

As already proven by a number of studies (Kerr et al. 1991; Meikle et al. 2004; Wathes et al. 2007), the multiparous cows in the present study produced a higher milk yield than the primiparous cows, regardless of their feeding. When considering only the multiparous, no effect of feeding on milk yield and ECM yield could be proven in the current study, whereas the milk yield in the 2P primiparous tended to be lower than in the 1P primiparous, and the ECM yield was lower in the 2P primiparous. Milk yield and milk composition determine predominantly profitability of dairy farming. McNamara et al. (2003) found that increased energy content in the precalving diet (4 week prepartum) led to higher milk, milk fat and milk protein yield in Holstein cows during the first 8 week of their second lactation. On the contrary, ad libitum feeding did not reveal any advantages over restricted feeding in terms of milk yield during early lactation in multiparous Holstein cows (Holcomb et al. 2001), and different energy levels in the prepartal ration also did not show any effect on milk yield (Doepel et al. 2002; Rabelo et al. 2003; Graugnard et al. 2012). With regard to primiparous, however, it should be considered that especially in the second half of gestation, the development of udder tissue takes place (Bruckmaier 2007). Therefore, the scarce supply of primiparous in group 2P with both energy and protein during this period (pasture and consequently low‐energy ‘Far off’ ration) may have had a negative effect on udder development and thus on p.p milk yield in this group.

Regarding milk composition, it was observed that milk urea in this study was very low, independently from feeding. In both, 1P multiparous (133 mg/L) and 2P multiparous (131 mg/L) the urea content in milk was below the reference range defined as 150–250 mg Urea/L by Fürll (2013). In combination with a milk urea content of < 150 mg/L, a milk protein content < 3.3% indicates insufficient energy and protein intake (Fürll 2013). This could indicate that the levels of NEL (6.9 MJ) and utilizable crude protein (158 g/kg DM) in the lactation diet were a little bit low. According to recommendations by NRC (2001), crude protein supply during early lactation should be 162 and 175 g/kg DM for primiparous and multiparous Holstein cows, respectively. This shows that protein supply might have been indeed a bit scarce, especially for multiparous cows, compared to NRC (2001).

## 5 Conclusions

The present study did not reveal clear advantages or disadvantages of either single‐phase or two‐phase feeding during the dry period. In addition, the timing of the shift to the ‘Close‐up’ ration appeared to be problematic, as the animals partially calved considerably earlier than the calculated date. The higher energy and protein intake of 2P multiparous compared to 1P multiparous ‘close‐up’ to parturition did not result in any advantages for the multiparous in terms of feed intake, milk yield and milk components or net energy balance during early lactation. Yet, serum NEFA concentrations tended to be higher in the 1P multiparous than in the 2P multiparous at the d of calving, presumably due to stimulated energy and nutrient intake during the ‘Far off’ period. Still, the postulated advantages of the biphasic feeding regime, for example, for DMI, net energy balance and incidence of peripartal diseases did not arise in the present study. Furthermore, in the case of primiparous, the two‐phase feeding system resulted in lower ECM yield during early lactation. Therefore, the extra work for 2P feeding seems not be justified.

## Ethics Statement

The authors confirm that the ethical policies of the journal, as noted on the journal's author guidelines page, have been adhered to and the appropriate ethical review committee approval has been received. The authors confirm that they have followed EU standards for the protection of animals used for scientific purposes. All animal procedures were carried out in accordance with ethical guidelines for the use of animal samples as approved by the State Office for Nature, Environment and Consumer Protection of the State of North Rhine‐Westphalia (LANUV; reference: 84‐02.04.2017.A096) and were carried out in accordance with the German Animal Welfare Legislation.

## Conflicts of Interest

The authors declare no conflicts of interest.

## Supporting information

Supplementary Material.

## Data Availability

The data that supports the findings of this study are available in the Supporting Information of this article.
