# Supplementary material for: Exploring Two Different Feeding Strategies During Dry Period With Regard to Dry Matter Intake, Intermediary Metabolism and Certain Health Aspects of Dairy Cows in Germany
Source: J Anim Physiol Anim Nutr (Berl). 2025 Apr 22;109(5):1073–90. doi: 10.1111/jpn.14120 (PMC12451420; doi:10.1111/jpn.14120)
Supplement: Supplementary file 1 — Supplementary Material. [file JPN-109-1073-s001.docx]

**Supplementary Material**

**Table S1.** Length of dry period (cows; mean ± SD) and preparation phase (heifers, mean ± SD), respectively, depending on feeding regime (single- vs. two-phase)

| Phase | Cows | | | Heifers | | |
| --- | --- | --- | --- | --- | --- | --- |
|  | 1P | 2P | *P*-Value | 1P | 2P | *P*-Value |
| Dry / preparation total | 51.4 ± 22.7 | 49.1 ± 14.7 | 0.65 | 51.6 ± 4.00 | 49.3 ± 6.01 | 0.18 |
| “Far off” dry / preparation |  | 33.6 ± 15.3 |  |  | 37.9 ± 3.33 |  |
| “Close up” dry / preparation |  | 15.4 ± 6.26 |  |  | 11.3 ± 4.10 |  |

1P, single-phase feeding. 2P, two-phase feeding. *P*-Value < 0.05 indicates significant difference between 1P and 2P.

**Table S2.** Dry matter intake (kg DM/d) of cows and heifers depending on feeding phase in the dry period (single- vs. two-phase); results from multivariable linear model approach.

| Lactation type | Examination period | 1P LSM | n | 2P LSM | n | SED | *P*-value |
| --- | --- | --- | --- | --- | --- | --- | --- |
| Cow | Early dry period | 15.2 | 46 | 12.4 | 45 | 0.40 | <0.001* |
|  | Late dry period | 13.9 | 46 | 14.4 | 45 | 0.45 | 0.2702 |
|  | Total dry period | 14.8 | 46 | 12.9 | 45 | 0.37 | <0.001* |
|  | Calving day | 10.4 | 46 | 9.96 | 44 | 0.97 | 0.670 |
|  | Lactation period | 22.0 | 46 | 22.4 | 45 | 0.63 | 0.509 |
| Heifer | Early preparation period | 12.4 | 17 | 9.69 | 18 | 0.53 | <0.001* |
|  | Late preparation period | 11.5 | 17 | 10.7 | 18 | 0.47 | 0.102 |
|  | Total preparation period | 12.0 | 17 | 9.90 | 18 | 0.48 | 0.001* |
|  | Calving day | 10.1 | 17 | 9.11 | 18 | 1.42 | 0.481 |
|  | Lactation period | 17.5 | 17 | 18.5 | 18 | 0.66 | 0.151 |

*Indicates a significant difference between the two treatments within each lactation type. 1P: single-phase feeding; 2P: two-phase feeding. LSM = least square means, n = Number of biological replicates, SED = standard error of the difference, *P*-Value for t-test of the difference. Total dry period = 6-8 wk a.p.; Early dry period = 4-6 wk; Late dry period = 2 wk a.p.

**Table S3.** Daily rumination time (min) of cows and heifers for various periods depending on feeding (single- vs. two-phase); results from multivariable linear model approach.

| Lactation type | Examination period | 1P LSM | n | 2P LSM | n | SED | *P*-value |
| --- | --- | --- | --- | --- | --- | --- | --- |
| Cow | Early dry period | 606 | 19 | 587 | 17 | 14.9 | 0.202 |
|  | Late dry period | 591 | 19 | 524 | 17 | 18.9 | 0.001* |
|  | Total dry period | 603 | 19 | 574 | 17 | 14.1 | 0.046* |
|  | Calving day | 329 | 18 | 289 | 15 | 33.5 | 0.243 |
|  | Lactation period | 616 | 19 | 609 | 16 | 16.5 | 0.666 |
| Heifer | Early reparation period | 574 | 6 | 669 | 6 | 72.9 | 0.224 |
|  | Late preparation period | 491 | 6 | 572 | 6 | 84.6 | 0.367 |
|  | Total preparation period | 558 | 6 | 636 | 6 | 67.1 | 0.273 |
|  | Calving day | 293 | 6 | 406 | 5 | 112 | 0.423 |
|  | Lactation period | 575 | 5 | 562 | 6 | 57.2 | 0.826 |

*Indicates a significant difference between the two treatments within each lactation type with regard to sampling time. 1P: single-phase feeding; 2P: two-phase feeding. LSM = least square means, n = Number of biological replicates, SED = standard error of the difference, *P*-Value for t-test of the difference. Total dry period = 6-8 wk a.p.; Early dry period = 4-6 wk; Late dry period = 2 wk a.p.
